# A Review on Composite Liposomal Technologies for Specialized Drug Delivery

**DOI:** 10.1155/2011/939851

**Published:** 2011-02-08

**Authors:** Maluta S. Mufamadi, Viness Pillay, Yahya E. Choonara, Lisa C. Du Toit, Girish Modi, Dinesh Naidoo, Valence M. K. Ndesendo

**Affiliations:** ^1^Department of Pharmacy and Pharmacology, University of the Witwatersrand, 7 York Road, Parktown, Johannesburg 2193, South Africa; ^2^Department of Neurology, University of the Witwatersrand, 7 York Road, Parktown, Johannesburg 2193, South Africa; ^3^Department of Neurosurgery, University of the Witwatersrand, 7 York Road, Parktown, Johannesburg 2193, South Africa

## Abstract

The combination of liposomes with polymeric scaffolds could revolutionize the current state of drug delivery technology. Although liposomes have been extensively studied as a promising drug delivery model for bioactive compounds, there still remain major drawbacks for widespread pharmaceutical application. Two approaches for overcoming the factors related to the suboptimal efficacy of liposomes in drug delivery have been suggested. The first entails modifying the liposome surface with functional moieties, while the second involves integration of pre-encapsulated drug-loaded liposomes within depot polymeric scaffolds. This attempts to provide ingenious solutions to the limitations of conventional liposomes such as short plasma half-lives, toxicity, stability, and poor control of drug release over prolonged periods. This review delineates the key advances in composite technologies that merge the concepts of depot polymeric scaffolds with liposome technology to overcome the limitations of conventional liposomes for pharmaceutical applications.

## 1. Introduction

Over the past few decades, liposomes have received widespread attention as a carrier system for therapeutically active compounds, due to their unique characteristics such as capability to incorporate hydrophilic and hydrophobic drugs, good biocompatibility, low toxicity, lack of immune system activation, and targeted delivery of bioactive compounds to the site of action [1–4]. Additionally, some achievements since the discovery of liposomes are controlled size from microscale to nanoscale and surface-engineered polymer conjugates functionalized with peptide, protein, and antibody [5, 6]. Although liposomes have been extensively studied as promising carriers for therapeutically active compounds, some of the major drawback for liposomes used in pharmaceutics are the rapid degradation due to the reticuloendothelial system (RES) and inability to achieve sustained drug delivery over a prolonged period of time [7]. New approaches are needed to overcome these challenges. Two polymeric approaches have been suggested thus far. The first approach involves modification of the surface of liposomes with hydrophilic polymers such polyethylene glycol (PEG) while the second one is to integrate the pre-encapsulated drug-loaded liposomes within depot polymer-based systems [3]. A study conducted by Stenekes and coworkers [8] reported the success of using temporary depot of polymeric materials to control the release of the loaded liposomes for pharmaceutical applications. This achievement leads to new applications, which requires collaborative research among pharmaceuticals, biomaterials, chemistry, molecular, and cell biology. Numerous studies in this context have been reported in the literature dealing with temporary depot delivery system to control the release of pre-encapsulated drug-loaded liposomes [9–12]. This system was developed to integrate the advantages while avoid the disadvantages of both liposome-based and polymeric-based systems. The liposome-based systems are known to possess limitations such as instability, short half-life, and rapid clearance. However, they are more biocompatible than the polymer-based systems [13]. On other hand, the polymer-based systems are known to be more stable and provide improved sustained delivery compared to liposome-based systems. However, one of the major setbacks is poor biocompatibility which is associated with loss of the bioactive (i.e., the drug) during fabricating conditions such as heat of sonication or exposure to organic solvents [3, 11]. The benefits of a composite system, however, include improvement of liposome stability, the ability of the liposome to control drug release over a prolonged period of time, and preservation of the bioactiveness of the drugs in polymeric-based technology. In addition, increased efficacy may be achieved from this integrated delivery system when compared to that of purely polymeric-based or liposome-based systems. The aim of this article therefore, is to review the current liposome-based and polymeric-based technologies, as well as the integration of liposome-based technology within temporary depot polymeric-based technology for sustained drug release. The discussion will focus on different types of liposome-based technology and depot polymeric scaffold technologies, various methods for embedding drug-loaded liposomes within a depot, and various approaches reported to control the rate of sustained drug release within depot systems over a prolonged period of time.

## 2. Liposome-Based Technology

**Figure 1:**
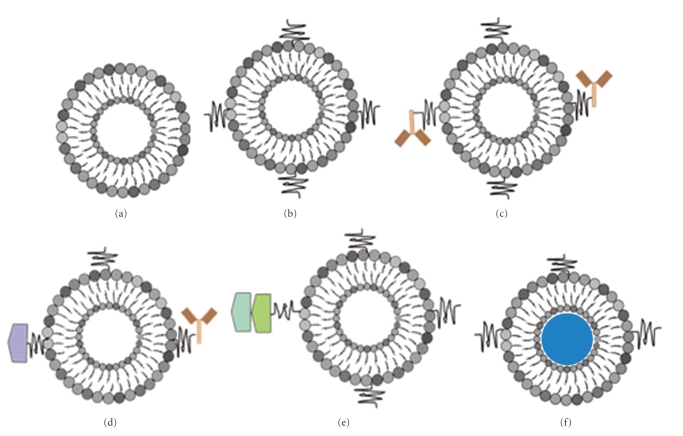
Schematic representation of liposome-based systems. (a) Conventional liposomes. (b) Stealth liposome coated with a polymeric conjugate such as PEG. (c) Stealth liposome coupled with a functionalized ligand. (d) Liposome with a single ligand and antibody. (e) Duplicated ligand with repeated peptide sequence. (f) Liposome loaded with perfluorocarbon gas (adapted from Zucker et al. [16]).

A liposome is a tiny vesicle consisting of an aqueous core entrapped within one or more natural phospholipids forming closed bilayered structures (Figure 1) [5]. Liposomes have been extensively used as potential delivery systems for a variety of compounds primarily due to their high degree of biocompatibility and the enormous diversity of structures and compositions [14, 15]. The lipid components of liposomes are predominantly phosphatidylcholine derived from egg or soybean lecithins [15]. Liposomes are biphasic a feature that renders them the ability to act as carriers for both lipophilic and hydrophilic drugs. It has been observed that drug molecules are located differently in the liposomal environment and depending upon their solubility and partitioning characteristics, they exhibit different entrapment and release properties [15, 16]. Lipophilic drugs are generally entrapped almost completely in the lipid bilayers of liposomes and since they are poorly water soluble, problems like loss of an entrapped drug on storage are rarely encountered. Hydrophilic drugs may either be entrapped inside the aqueous cores of liposomes or be located in the external water phase. Noteworthy is that the encapsulation percentage of hydrophilic drugs by liposomes depends on the bilayer composition and preparation procedure of the liposomes [17, 18].

Since liposome discovery by Bangham and coworkers [5], several different embodiments of liposome-based technology have been developed to meet diverse pharmaceutical criteria [7]. Liposome-based technology has progressed from the first generation “conventional vesicles,” to stealth liposomes, targeted liposomes, and more recently stimuli-sensitive liposomes [3, 19]. Essentially, liposomes are classified according to their size range, being 50–5000 nm in diameter. This resulted into two categories of liposomes namely multilamellar vesicles and unilamellar vesicles [19]. Unilamellar vesicles consist of single bilayer with a size range of 50–250 nm while multilamellar vesicles consist of two or more lipid bilayers with a size range of 500–5000 nm [3, 20].

### 2.1. Conventional Liposomes

Conventional liposome-based technology is the first generation of liposome to be used in pharmaceutical applications [3, 21, 22]. Conventional liposome formulations are mainly comprised of natural phospholipids or lipids such as 1,2-distearoryl-sn-glycero-3-phosphatidyl choline (DSPC), sphingomyelin, egg phosphatidylcholine, and monosialoganglioside. Since this formulation is made up of phospholipids only, liposomal formulations have encountered many challenges; one of the major ones being the instability in plasma, which results in short blood circulation half-life [7, 23–25] Liposomes that are negatively or positively charged have been reported to have shorter half-lives, are toxic, and rapidly removed from the circulation [23, 26, 27]. Several other attempts to overcome these challenges have been made, specifically in the manipulation of the lipid membrane. One of the attempts focused on the manipulation of cholesterol. Addition of cholesterol to conventional formulations reduces rapid release of the encapsulated bioactive compound into the plasma [28]. Furthermore, studies by Tran and coworkers [29] demonstrated liposome stability after addition of “helper” lipids such as cholesterol and 1,2-dioleoyl-*sn*-glycero-3-phosphoethanolamine (DOPE). Harashima and coworkers [20] demonstrated that phagocytosis of liposomes was due to the size of the liposome formulation. Larger size or multilamellar liposomes with a size range of 500–5000 nm were the first to be eliminated from the systemic circulation. Nanosized liposomes or small unilamellar vesicles with a size range of 20–50 nm were only developed later [7, 20, 30]. The following drugs: Ambisone, Myocet, Daunoxome, and Daunorubicin have received clinical approval using conventional liposome technologies [31–33]. Although small unilamilar liposomes were reported to have potential for a decreased microphage uptake, insufficient drug entrapment is still a major disadvantage. On the basis of this study, the success of cholesterol and others phospholipids did not completely overcome the major challenges.

### 2.2. Stealth Liposomes

**Figure 2:**
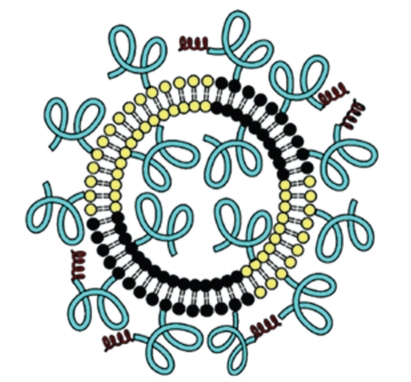
Schematic depicting of a stealth PEGylated liposome (Adapted from Rai et al. [58]).

Stealth liposome technology is one of the most often used liposome-based systems for delivery of active molecules [3, 22]. This strategy was developed to overcome most of the challenges encountered by conventional liposome technology such as the inability to evade interception by the immune system, toxicity due to charged liposomes, low blood circulation half-life, and steric stability [7, 22, 26]. Stealth liposome strategy was achieved simply by modifying the surface of the liposome membrane, a process that was achieved by engineering hydrophilic polymer conjugates [34]. The employed hydrophilic polymers were either natural or synthetic polymers such polyethylene glycol (PEG), chitosan, silk-fibroin, and polyvinyl alcohol (PVA) [35–38]. Several properties that would add advantages to polymeric conjugate were considered such as high biocompatibility, nontoxicity, low immunogenicity, and antigenicity [3, 35]. Although the majority of hydrophilic polymers meet the above criteria, PEG remains the most widely used polymer conjugate. It is specifically employed to increase the hydrophilicity of the liposome surface via a cross-linked lipid [39, 40]. PEGylated liposomal doxorubicin (DOXIL/Caelyx) is the exceptional example of stealth liposome technology to be approved by both the USA Food and Drug Administration (FDA) and Europe Federation [41]. Although prominent results were achieved from this model such as reduction of macrophage uptake, long circulation, and low toxicity, passive targeting is still a major disadvantage since liposomes can deliver active molecules not only to abnormal cells but also to sensitive normal cells [7, 42].  Figure 2 depicts a schematic for a PEGylated liposome.

### 2.3. Targeted Liposomes

Targeted liposome based system was suggested after conventional stealth liposome failed to evade uptake of active molecules by sensitive normal cells or nonspecific targets *in vivo* [43, 44]. Unlike stealth liposome, site-specific targeting liposome has been engineered or functionalized with different types of targeting moieties such antibodies, peptide, glycoprotein, oligopeptide, polysaccharide, growth factors, folic acid, carbohydrate, and receptors [45–50]. In addition, targeted ligand can further increase the rate of liposomal drug accumulation in the ideal tissues/cells via overexpressed receptors, antigen, and unregulated selectin [51–55]. Peptides, protein, and antibodies have been most extensively studied as a ligand for directing drug-loaded liposomes into sites of action, due to their molecule structures, which are essentially composed of known amino acid sequences. Furthermore, it has been postulated that ligands can be conjugated onto pegylated liposomes via different types of coupling methods, such as covalent and noncovalent binding. Covalent coupling occurs when novel ligands are indirectly engineered on the surface of liposome through a hydrophobic anchor via thioether, hydrazone bonds, avidin–biotin interaction, cross-linking between carboxylic acids and/or amines [56]. Noncovalent coupling is observed when novel ligands are directly added to the mixture of phospholipids during the liposomal formulation [15]. Li et al. [48] attempted to generate dual ligand liposome conjugates aimed at targeting multiple receptor types on the cell surface receptors. *Ex vivo* studies demonstrated the success of the dual ligand approach in improving the selectivity when compared to a single ligand approach. In another study, Ying and coworkers [50] formulated dual targeted liposomes with various targeted moieties such as p-aminophenyl-*α*-D-manno-pyranoside (MAN) and transferrin (TF). The study was conducted both *ex vivo* (in C6 glioma cells) and *in vivo* (in C6 brain glioma-bearing rats). The following were compared: free daunorubicin, daunorubicin liposomes, daunorubicin liposomes modified with MAN, and daunorubicin liposomes modified with TF as the controls, and daunorubicin liposomes modified with MAN and TF. Daunorubicin liposomes modified with dual ligands such as MAN and TF showed a more significant increase in therapeutic efficacy, when compared with the drug alone, drug-loaded liposome, or single ligand modified surface of the liposome. However, the efficacy of these approaches faces limitations because protein circulation and gene expression cannot be sustained for long periods of time [7]. Doxorubicin-loaded liposomes were surface engineered with monoclonal antibody and are now commercially available [57]. The overall advantage of this model of liposome is an increase in active molecules or drug reach targeted cells via endocytosis [7].

**Figure 3:**
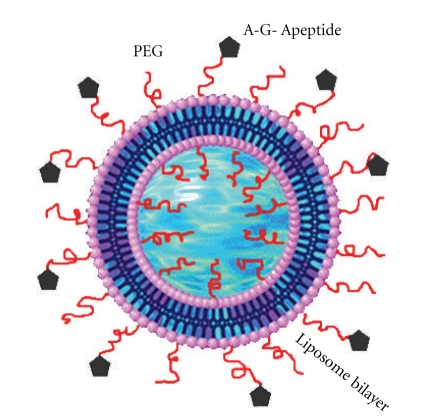
A schematic representation of the targeted liposome delivery system depicting the cyclic RGD peptides that targets the *α*_v_ *β*_3_ integrin receptors on the vascular tumor cells (adapted from Nallamothu et al. [59]).

In another study, Nallamothu and coworkers [59] demonstrated the usefulness of Combretastatin A4 as novel antivascular agent. This compound portrays its anticancer activity by inducing irreversible vascular shutdown in solid tumors [60]. Despite its anticancer potential, the drug has shown to have several undesirable side effects to the underlying normal tissues [61]. These problems may be alleviated by targeting the drug specifically to the solid tumor vasculature. Studies have shown that certain cell adhesion molecules such as *α*_v_ *β*_3_ integrin receptors are overexpressed on actively proliferating endothelium of the tumor vasculature [62, 63]. These surface markers discriminate tumor endothelial cells from the normal endothelial cells and can be used as a target for antivascular drug delivery [59]. Nallamothu and coworkers [59] could demonstrate that peptides with Arginine-Glycine-Aspartine (A-G-A) amino acid sequence constrained in a cyclic polyethylene-glycol (PEG)-based liposome framework can bind to the *α*_v_ *β*_3_ integrin receptors. Basing on this analogy, they could design a targeted liposome delivery system for combretastatin A4 with cyclic (RDG) peptides as targeting ligands (Figure 3). Targeting of combretastatin A4 to irradiated tumors using this delivery system resulted into significant tumor growth delay [59].

### 2.4. Other Types of Liposomes

#### 2.4.1. Virosomes and Stimuli-Responsive Liposomes

**Figure 4:**
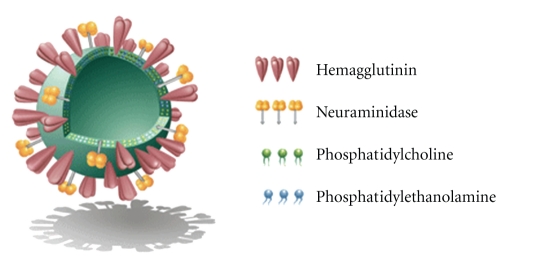
A schematic representation of a virosome (source: Pevion Biotech Ltd. [73]).

Liposomal technologies, such as conventional, stealth, and targeted liposomes have already received clinical approval [64, 65]. New generation types of liposome have been developed to increase bioactive molecule delivery to the cytoplasm by escape endosome [1, 66, 67]. New approaches that employ liposomes as pharmaceutical carriers are virosomes and stimuli-type liposomes. The stimulating agents in this case include pH, light, magnetism, temperature, and ultrasonic waves. A virosome (Figure 4) is another type of liposome formulation. It comprises noncovalent coupling of a liposome and a fusogenic viral envelop [68]. A stimuli-sensitive liposome is a type of liposome that generally depends on different environmental factors in order to trigger drug, protein, and gene delivery. A study conducted by Schroeder et al. [69], Liu and coworkers [67], and Lentacker and coworkers [70] demonstrated that the exposure of the liposome loaded with perfluorocarbons gas to ultrasound waves triggered drug and gene delivery into the cytoplasm of the targeted cells through cell membrane pores. Their data demonstrated that the liposome-loaded magnetic agents triggered drug delivery to the specific site *in vivo*, using an externally applied magnetic field. The enhancement of endosomal release of drug-loaded liposome into the cytoplasm was also reported to be influenced by the utilization of pH-sensitive liposomes or by attachment of pH-sensitive fusogenic peptide ligands [1, 71, 72]. Most recently, a review article published by Chen and coworkers [4] described the generation of stable liposomes utilizing lyophilization techniques, which may be a promising future model for liposome production.

#### 2.4.2. Gene-Based Liposomes

**Figure 5:**
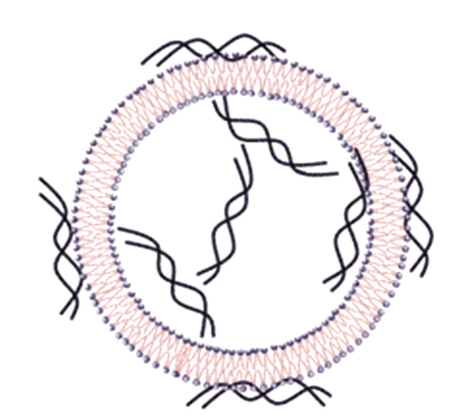
A schematic representation of a DNA-liposome complex (adapted from Uchegbu [74]).

The characterization of human genome coupled with recombinant DNA technology has created opportunities for gene therapy that never existed before [74]. Candidate diseases for such technology include cancer [75], arteriosclerosis [76], cystic fibrosis [77], haemophilia, sickle cell anaemia, and other genetic diseases. Ideally, the administration of the gene of interest should result in the expression of the therapeutic protein. However, the delivery of the large anionic bioactive DNA across cell has been one of the most difficult endeavors. DNA is easily degraded by circulating and intracellular deoxyribonucleases. Notwithstanding, it must also be delivered intact across the cell and nucleolar membranes to the nucleus [74]. Liposomes have thus proved to achieve efficient intracellular delivery of DNA [78, 79]. Such liposomes are prepared from phospholipids with an amine hydrophilic head group. The amines may be either quaternary ammonium, tertiary, secondary, or primary, and the liposomes prepared in this way are commonly referred to as cationic liposomes, since they possess a positive surface charge at physiological pH. The use of cationic liposomes as gene delivery systems was firstly enforced in the late 1980s when *in vitro* studies by Felgner and coworkers [80] could demonstrate that the complexation of genes with liposomes may promote gene uptake by cells *in vitro*. Since then, cationic liposomes of varying description have been used to promote the cellular uptake of DNA with resultant therapeutic protein expression by various organs in vivo.  Figure 5 depicts a schematic representation of a DNA-liposome complex.

**Figure 6:**
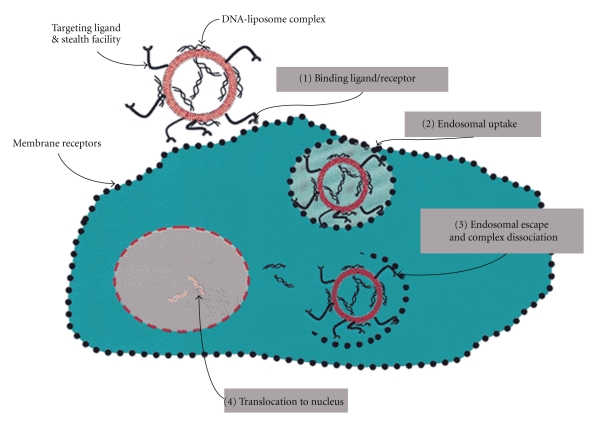
A schematic depicting the optimization of liposomal gene delivery (source: Uchegbu [74]).

Although the experimental data have demonstrated that cationic liposomes can facilitate the transfer of DNA into live mammalian cells, there are still major problems that need to be overcome in order to effectively achieve the goal. These include a reduction in the rapid clearance of cationic liposomes and the production of efficiently targeted liposomes. At the cellular level, the problems may be overcome by improving receptor mediated uptake employing appropriate ligands. The endowment of liposomes with endosomal escape mechanisms, coupled with more efficient translocation of DNA to the nucleus and the efficient dissociation of the liposome complex just before the entry of free DNA into the nucleus might provide an optimal cornerstone solution to the problem. This proposition is depicted in Figure 6. 

## 3. Temporary Depot Polymeric-Based Systems for Liposomal Coupling

Polymer-based systems, such as hydrogel or prefabricated scaffolds have been used as depots for drugs, regenerative cells, protein, growth factor, and pre-encapsulated drug-loaded liposome for sustained release [8, 12, 81–85]. Various polymers have been researched for this application based on their fundamental properties such as biodegradability, biocompatibility, nontoxicity, and the noninflammatory tendency. Natural and synthetic biodegradable polymeric systems such chitosan, collagen, gelatin, fibrin, alginate, dextran, carbopol, and polyvinyl alcohol have been employed as temporary depot-forming agents since they meet most of the above requirements [11, 84, 86, 87].

### 3.1. Injectable Polymeric Scaffolds

**Figure 7:**
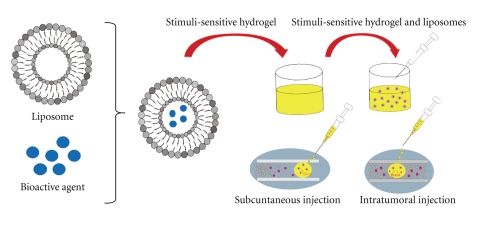
Schematic depicting drug delivery from pre-encapsulated drug-loaded liposomes incorporated within an injectable hydrogel-based system (adapted from Ta et al. [93]).

The strategy for generating an ideal depot for an active compound or bioactive molecule-loaded liposome with the benefit of in local drug retention and sustained release over prolonged time has recently received much attention in both pharmaceutical and bioengineering research [85, 88]. The *in-situ* forming injectable polymer was among the most successful models, since it was able to encapsulate protein and/or bioactive molecules or function as a pre-encapsulated drug-loaded liposomal formulation that was in liquid form [89, 90]. This solution or suspension mixture could then be injected into the target organ with a needle to form a semisolid scaffold and finally an implant. The success in shifting from liquid formulation to semisolid and finally to an implant was a result of various desirable polymeric properties and stimulating agents such as water, light, temperature, and pH, that facilitated such processes within the polymer such as precipitation, cross-linking, and polymerization [88, 91–93]. Since the majority of hydrogels were composed of natural or synthetic biodegradable polymers, bioactive molecules were released via passive diffusion, matrix pore formation, or polymeric degradation [94–97]. Furthermore, semisolid implant formation was reported as being dependant on the polymeric state such as phase inversion, low-glass transition temperature, or on hydrogels that formed by the aid of cross-linking reagents and chemo- or thermosensitazation [98, 99]. In addition, the system could deliver drug directly or indirectly to the targeted sites, through subcutaneous injection and/or intratumoral injection (Figure 7) [93]. Overall, the semisolid temporary depots offer several advantages such as enhanced local drug retention, sustained release, and potential for long-term storage. However, repeated injections and passive drug release are still a factor that limits their use as ideal pharmaceutical carriers. 

### 3.2. Prefabricated Polymeric Scaffolds

**Figure 8:**
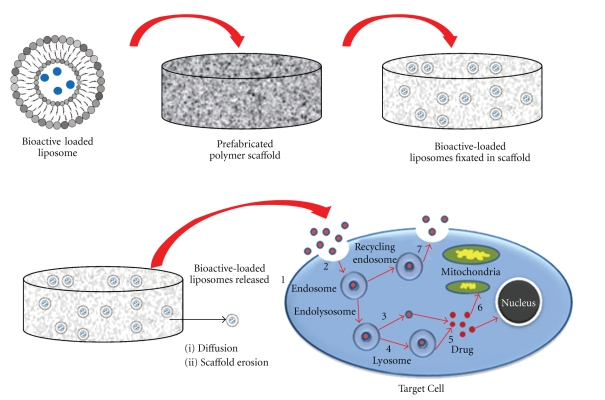
Schematic depicting drug delivery from pre-encapsulated drug-loaded liposomes incorporated within a prefabricated polymeric-based depot system with eventual entry through a cell membrane (adapted from Stenekes et al. [8]).

Prefabricated polymeric scaffolds have gained a lot of attention as depots for delivery of bioactive molecules, regenerative cells, growth factors, and pre-encapsulated bioactive loaded liposome [100, 101]. Unlike injectable *in situ* scaffolds in which a semisolid scaffold is achieved after injection, prefabricated polymer scaffold solid depot materials are formed outside the body, then surgically implanted [102]. In additional, pre-fabrication polymeric scaffold can be designed to meet the required characteristics of an ideal scaffold. Desirable attributes of an ideal scaffold are: three-dimensional structure, appropriate surface chemistry, fabrication from materials which are biodegradable or bioresorbable, should not induce any adverse response, scaled pore capacity, and highly reproducible shapes and size [99, 101]. Different fabrication techniques have been used to achieve the above criteria, such as fiber bonding, emulsion freeze drying, solvent casting, high-pressure processing, gas foaming, and electrospinning [102–105]. Various polymers that have been researched for this application are either biodegradable or nondegradable, synthetic or natural, or a combination of the two [9, 106]. The major challenge of prefabricated polymeric scaffolds is that a nondegradable polymeric device requires surgical removal at the end of treatment, which is often known to be associated with pain [107]. However, the benefit on sustained release for the pre-encapsulated drug loaded scaffold over a long period of time has been reported and declared successful [96]. Stenekes and coworkers [8] demonstrated that liposome embedded inside a biodegaradble depot polymeric scaffold was able to sustained drug release over a prolonged period of time (Figure 8). In addition, the released liposome was found intact after many days storage within the inside depot polymeric scaffold.

## 4. Natural Product-Based Liposomal Drug Delivery Systems

### 4.1. Collagen-Based Liposomal Drug Delivery Systems

**Figure 9:**
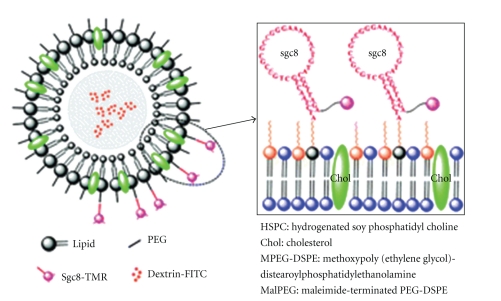
A schematic representation of a collagen-based liposome (source: Kang et al. [121]).

Collagen is a major natural protein component in mammals that is fabricated from glycine-proline-(hydroxy) proline repeats to form a triple helix molecular structure [84]. So far, nineteen types of collagen molecules have been isolated, characterized, and reported in both medical and pharmaceutical applications [108–110]. Collagen has been widely used in pharmaceutical applications due to the fulfillment of many requirements of a drug delivery system such as good biocompatibility, low antigenicity, and degradability upon implantation [111]. Furthermore, collagen gels are one of the first natural polymers to be used as a promising matrix for drug delivery and tissue engineering [112]. Biodegradable collagen-based systems have served as 3D scaffold for cell culture, survival of transfected fibroblasts, and gene therapy [81, 113]. In this case, collagen scaffolds were fabricated through introducing various chemical cross-linking agents (i.e., glutaraldehyde, formaldehyde, carbodiimide) or by physical treatments (i.e., UV irradiation, freeze-drying, and heating) [109, 114–117]. The combination of liposomes and collagen-based technologies has been long achieved since the early 80s [112]. In this case, drugs and other bioactive agents were firstly encapsulated in the liposomes and then embedded inside a depot composed of collagen-based systems, including scaffolds and gels. The combination of these two technologies (i.e., liposomes and collagen-based system) has improved storage stability, prolonged the drug release rate, and increased the therapeutic efficacy [84, 118, 119]. In addition, a study that was conducted by Marston et al. [120], demonstrated that temperature sensitive liposomes and collagen may thermally trigger the release of calcium and phosphate salts. Multiple collagen-based system for pharmaceutical carriers or medicinal applications are currently available for clinical purposes [121].  Figure 9 depicts a schematic representation of collagen-based liposome.

### 4.2. Gelatin-Based Liposomal Drug Delivery Systems

Gelatin is a common natural polymer or protein which is normally produced by denaturing collagen [122]. It has been used in pharmaceutical and medical applications due to its outstanding properties such as biodegradability, biocompatibility, and low antigenicity [100]. In addition, gelatin can be easy to manipulate due to its isoelectric point that allows it to change from negative to positive charge in an appropriate physiological environment or during the fabrication, a property that has found it being very attractive to many pharmaceutical researchers [123]. Gelatin is one of the natural polymers used as support material for gene delivery, cell culture, and more recently tissue engineering. Gelatin-based systems have the ability to control release of bioactive agents such as drugs, protein, and dual growth factors [95, 100, 124]. It has been reported that it is possible to incorporate liposome-loaded bioactive compounds into PEG-gelatin gel which function as porous scaffold gelatin-based temporary depots with controlled drug release over prolonged periods of time [125, 126]. However, some setbacks have been identified, and they are said be associated with the use of gelatin-based systems in pharmaceutical applications. These setbacks include poor mechanical strength and ineffectiveness in the management of infected sites [108]. A combination of a collagen-based system with liposomes has been proposed to achieve the stability of the system and controlled release profiles of the incorporated compounds. The success of these formulations, (i.e., gelatin, hydrogel, and scaffolds) was enhanced by cross-linking agents such as glutaraldehyde, sugar, and enzyme transglutaminase. It was also discovered that the cross-linking density of gelatin was able to affect the rate of degradation and rate of bioactive agents release from gelatin vehicles or from liposomes embedded inside gelatin-based systems [127–130]. Another study by Peptu and coworkers [83] reported a controlled release of liposome-encapsulated calcein fluoroscence dye or calcein labeled with rhodamine from temporary depot of gelatin-based system which is made up of Gelatin-carboxymethylcellulose films. In the same study, the release rate of loaded liposome was found to depend mostly on the quantity of liposomes entrapped inside the films, degree of swelling of the film, film network density, and the film geometry which was supported by glutaraldehyde cross-linking agents. In a similar study, DiTzio and coworkers [125] demonstrated the success of prevention of bacterial adhesion to catheters by ciprofloxacin-loaded liposomes which were entrapped inside a poly(ethylene glycol-)gelatin hydrogel. Another study by Burke and coworkers [126] demonstrated that there was a successive release of oxidizing reagent (sodium periodate) from thermal liposome entrapped inside a stimuli-responsive gelatinous derivative hydrogel. In general, the combination of collagen with liposome has been reported to improve liposome stability and the controlled release of incorporated bioactive agents within liposome formulations.

### 4.3. Chitosan-Based Liposomal Drug Delivery Systems

Chitosan is a natural linear bio-polyaminosaccharide polymer obtained by *N-*deacetylation of chitin, which is fabricated from the exoskeleton of marine crustaceans such as shrimps, crabs, prawns, and fungi [87, 131]. It has been broadly investigated in pharmaceutical applications as a bioactive molecule delivery method or as depot of pharmaceutical carriers due to its desirable properties such as mucoadhesiveness, biodegradability, biocompatibility, and nontoxicity [132–135]. The combination of chitosan with liposome technologies is considered as being a promising approach in the drug delivery arena. More recently, chitosan technology has been reported as being a depot for liposomal drug delivery systems in the form of porous hydrogel or scaffold. Chitosan-based hydrogels were generate with or without a cross-linking agent such as glutaraldehyde or by interacting with different types of divalent and polyvalent anions [12, 136, 137]. Novel *in situ *gelling formulations of hydrogels such as thermosensitive and mucobioadhesive hydrogels have been recently been proposed as a depot for liposomes for sustained drug release over a prolong period of time [12, 138]. Chitosan scaffold matrix can be fabricated with unique structure by simple approaches such lyophilization technique, by use of crosslinked agents of chitosan solution/hydrogels followed by incubation in the liquid nitrogen, or by employing liquid carbon dioxide, solid-liquid separation, and, most recently, supercritical immersion precipitation techniques [11, 139–141]. Drugs such as cytarabine that have been pre-encapsulated in liposomes and then incorporated within chitosan hydrogels have been proven to be suitable model for drug delivery with sustained drug release *in vivo* at body temperature [12].

### 4.4. Fibrin-Based Liposomal Drug Delivery Systems

Fibrin is a biodegradable polymer obtained by polymerization of fibrinogen in the presence of thrombin enzyme [142]. The concept of developing fibrin-based technology as a temporary depot in both pharmaceutical and bioengineering fields has received considerable attention over the past decades [82, 143]. The unique properties of the fibrin-based systems such biodegradability and nontoxicity, have been reported to influence the delivery efficiency of growth factors, genes, proteins, various cells and drugs [144–150]. The fabrication of semirigid fibrin scaffold upon injection has been achieved under physiological conditions at the site of interest with rapid polymerization [147]. Furthermore, fibrin scaffolds have also been used as temporary depots for drug delivery vehicles by incorporation of drug-loaded liposomes alone, or by incorporation of liposomes into a chitosan matrix (containing bioactive agent molecules such as protein, drugs and genes) within the depot composed of the fibrin-based systems. The combination of two widespread devices, fibrin and liposome technologies, resulted in sustained bioactive agent release over prolonged periods of time [11, 146, 150–152].

### 4.5. Alginate-Based Liposomal Drug Delivery Systems

Alginate also serves as an example of a naturally occurring linear polysaccharide. It is extracted from seaweed, algae, and bacteria [153–155].The fundamental chemical structure of alginate is composed of (1–4)-b-D-mannuronic acid (M) and (1–4)-a-L-guluronic acid (G) units in the form of homopolymeric (MM- or GG-blocks) and heteropolymeric sequences (MG or GM-blocks) [156]. Alginate and their derivates are widely used by many pharmaceutical scientists for drug delivery and tissue engineering applications due to its many unique properties such as biocompatibility, biodegradability, low toxicity, non-immunogenicity, water solubility, relatively low cost, gelling ability, stabilizing properties, and high viscosity in aqueous solutions [157, 158]. Since alginate is anionic, fabrication of alginate hydrogels has successively been achieved through a reaction with cross-linking agents such as divalent or trivalent cations mainly calcium ions, water-soluble carbodiimide, and/or glutaraldehyde [159]. The cross-linking methodology was conducted at room temperature and physiological pH [160]. The success in fabricating highly porous 3D alginate scaffolds has been through lyophilization [161]. Thus far, alginate-based systems have been successfully used as a matrix for the encapsulation of stem cells and for controlled release of proteins, genes, and drugs [162–166]. In addition, alginate-based systems have been used as depots for bioactive agent-loaded liposomes, for slow drug release [9, 167]. Highly increased efficacy has been reported from these integrated delivery systems when compared to polymeric-based systems or liposome-based systems alone [168, 169]. Machluf and coworkers [170] have reported radio labeled protein release from liposomes encapsulated within microspheres of the calcium-crosslinked alginate. Another study by Hara and Miyake [171] demonstrated the release of Calcein (which is a fluorescent dye) and Insulin from calcium alginate gel-entrapped large multilamellar liposomal vesicles *in vivo*.

### 4.6. Dextran-Based Liposomal Drug Delivery Systems

Dextran is a natural linear polymer of glucose linked by a 1–6 linked-glucoyranoside, and some branching of 1,3 linked side-chains [172]. Dextran is synthesized from sucrose by certain lactic-acid bacteria, the best-known being *Leuconostoc mesenteroides* and *Streptococcus mutans*. There are two commercial preparations available, namely dextran 40 kilodaltons (kDa) (Rheomacrodex) and dextran 70 Kilodaltons (kDa) (Macrodex) [173, 174]. In pharmaceutics, dextran has been used as model of drug delivery due to its unique characteristics that differentiate it from other types of polysaccharide. This include water solubility, biocompatibility, and biodegradability [175]. In recent studies, dextran has been regarded as a potential polysaccharide polymer that can sustain the delivery of both proteins, vaccines, and drugs [176–179]. Interleukin-2, which is a highly effective anticancer drug, is among the success obtained in delivering a combination of drug-loaded liposome and injectable dextran hydrogel [180]. Injectable and degradable dextran-based systems for drug delivery were generated by a cross-linking reaction with photo-polymerization or free radical polymerization [181]. In another study by Yeo and Kohane [182], it was demonstrated that it is possible to fabricate dextran-based hydrogel using dextran derivatives such as carboxymethyldextran derived by aldehyde-modification or carboxymethylcellulose. In the same study, dextran-based systems were reported to inhibit peritoneal adhesions due to cytotoxicity. Cytotoxicity study was demonstrated in mesothelial cells and macrophages, and it's reported to be associated with a crosslinked agent [182]. A study by Stenekes and coworkers [8] demonstrated the successive encapsulation of a drug-loaded liposome depot into a dextran polymer-based material. The polymeric-based materials were fabricated using a two phase system, the first phase was water and poly(ethylene glycol) and the second one water methacyrlated dextran. The slower degradation of dextran polymeric material resulted in sustained liposome release over a period of 100 days [8]. Liposomes released from depot were reported to be intact, and there was no significant change in liposomal size. In a gene therapy study by Liptay and coworkers [183], it was reported that recombinant DNA (which contains chloramphenicol acetyltransferase) was successively encapsulated in cationic liposomes and then integrated within dextran. This system was reported to be a suitable delivery system since it could stop transfection efficiency within the colon epithelium wall *in vivo *[183].

## 5. Liposomal Drug Delivery Systems Based on Synthetic Polymers

### 5.1. Carbopol-Based Liposomal Drug Delivery Systems

Carbopol hydrogel formulation is a synthetic type of hydrogel, which is a polyacrylic acid derivative. Carbopol 980, Carbopol 974NF resin, and Carbopol 940 have been widely used as pharmaceutical carriers due to their outstanding properties such as bioadhesivity, biocompatibility, and low toxicity [184–186]. Carbopol can swell quickly in water and adhere to the intestinal mucus because the functional carboxylic acid groups (–COOH) can form hydrogen bridges to interpenetrate the mucus layer [187, 188]. Furthermore, carbopol can inhibit the activity of the dominant enzymes in the gastrointestinal tract due to the possession of carboxylic groups in its structure [187]. In a study that was conducted by Tang and coworkers [186], the formulation of Carbopol containing superporous hydrogel composites showed that swelling was due to ionic strength in salt, sensitive at different pH values. In recent studies, Hosny [189, 190] reported the possibility of incorporating drug-loaded liposome within Carbopol hydrogel-based system which acted as a temporary depot. They conducted the study *in vitro *with the aim of improving low viscosity and poor sustainability release over a prolonged period of time, which is associated with liposome setbacks. The results suggested that the degree of encapsulation and prolongation of drug release rate of either drugs or loaded liposomes in temporary depots of Carbopol depends to a great extent on the properties of the vesicles, such as charge and rigidity. Various drugs such as ciprofloxacin and galifloxacin have been reported to have been employed in this system, by firstly being encapsulated within liposomes and then integrated within the temporary depot of the Carbopol-based system. These studies revealed that loaded liposome integrated within Carbopol-based system was a suitable model of drug delivery for both ocular and vaginal disorders [189–192].

### 5.2. Polyvinyl Alcohol-Based Liposomal Drug Delivery Systems

Polyvinyl alcohol (PVA) is a water soluble highly hydrophilic synthetic polymer, with a molecular mass of 80 killodaltons (KDa). PVA can be used in a widely range of applications such industrial, commercial, medical, and food products [193, 194]. In addition, PVA has gained a lot of attention in pharmaceutical applications due to some attractive properties such as low toxicity, excellent film-forming, biodegradability, emulsifying capacity, biocompatibility, and adhesive properties [195, 196]. PVA-based hydrogel or scaffolds have been fabricated using chemical cross-linking agents such as citric acid derivative, glutaraldehyde, and formaldehyde, or by physical cross-linking processes such as ultraviolet photo-cross-linking, freezing-thawing, and radiation [126, 197, 198]. Various studies have been performed on the effects of PVA-based polymers on the release rate of pre-encapsulated drug-loaded liposomes. In these combination systems, PVA was postulated to enhance liposome viscosity, making them more stable and less permeable, thus providing a sustained release liposomal delivery system [185]. A recent study conducted by Litvinchuk and coworkers [199] demonstrated that the success of calcein-loaded liposome embedded inside a temporary depot was influenced by photocross-linking. In the same study, the fluorescence intensity was reported to result in a sustained release effect as observed from day 0 to 120, in both phosphate buffer saline and blood plasma *in vitro*. Overall, the study demonstrated that PVA as a temporary depot offers several advantages to liposome delivery systems. These include liposome stability, viscosity, and sustained drug release over prolonged periods of time. Ciprofloxacin, a synthetic chemotherapeutic antibiotic was among the drugs that were reported to have been successfully integrated into liposome and PVA-based delivery systems [185].

## 6. Techniques for Embedding Drug-Loaded Liposomes within Depot Polymeric-Based Systems

Different techniques of loading the drug within temporary depot polymeric-based systems either by using natural or synthetic polymers have been reported by many researchers [8, 11, 12, 118, 185]. However, several disadvantages were found to be associated with this approach such as loss of the efficacy of the drugs during the fabrication process due to the acidic, basic, and/or toxic effect of the solvents employed, heat of sonication, or biochemical interactions with polymeric-based materials such human fibrin gel [11, 200]. To avoid these setbacks, new techniques were suggested by firstly pre-encapsulating the drugs within liposome and then embedding the drug-loaded liposome into the temporary depot polymeric-based system. This approach attracted many researchers as it improved drug delivery and at the same time preserved drug bioactivity [11, 36, 185, 189, 201]. The success of this technique was also reported after pre-encapsulating drug-loaded liposomes into fibrinogen solution, then injecting the mixture into porous chitosan films [11, 201]. Another approach using synthetic PVA was made in which thin films of liposomes were hydrated above their glass transition temperature together with PVA as the hydration solution in order to enhance liposome entrapment into the temporary depot of PVA-based system [185]. Thermosensitive hydrogel was also investigated using a chitosan derivative, which is temperature sensitive. In this case, drug-loaded liposome was mixed with prechilled solutions of chitosan solution until an iso-osmotic pressure was achieved within the chitosan solution [12]. In another study that was conducted by Gobin and coworkers [36], it was demonstrated that drug-loaded liposomes were incorporated within a polymeric-based system with agitation and subsequently lyophilized after being frozen overnight at −80°C. Tabandeh and Aboufazelia [118] suggested a nitrogen refrigeration approach. In this case, pre-encapsulated drug-loaded liposomes were mixed together with collagen solution and then frozen in liquid nitrogen for 24 hours. Since soluble collagen was used in this study, adequate concentrations of collagen were suggested in order to facilitate the drug release and avoid the chain mobility associated with collagen. 

A more recent study has demonstrated an enhanced process of drug-encapsulated liposome into Carbopol hydrogel by using deionized water as a vehicle (i.e., employing a hydration approach) [189]. This involved the development of an effective prolonged-release liposomal hydrogel formulation containing ciprofloxacin for ocular therapy. Drug delivery in ocular therapy has for long been a difficult task to accomplish because of the poor drug bioavailability that is mainly due to the precorneal loss factors. These factors include tear dynamics, insufficient residence time in the conjunctival sac, and nonproductive absorption [185, 202]. Thus far, fluoroquinolones have shown excellent activity against most of the frequently occurring Gram-positive and Gram-negative ocular pathogens [189]. Earlier generations of fluoroquinolones (e.g., ofloxacin) were often encountered with a problem of developing resistance at a fast rate [203, 204]. Ciprofloxacin is active against a broad spectrum of aerobic Gram-positive and Gram-negative bacteria. Resistance to this drug develops slowly and has shown to cause a minimal toxicity [189]. It is currently the drug of choice as an anti-infective ocular agent [205, 206]. Efficacy of the marketed ophthalmic fluoroquinolone products, mostly aqueous solutions, is limited by poor ocular bioavailability, compelling the frequent dosing regimen, and uncompromised patient compliance [207, 208]. Thus, prolonged-release ciprofloxacin liposomal hydrogel has proven to be a suitable delivery system for ocular infections.

## 7. Modulating Drug Release from Liposomes within Polymeric Depot Systems

Sustained release of therapeutically active compounds loaded with liposome in a depot incorporated into polymeric-based system offers the possibility of reducing the dosing frequency, which may lead to the reduction of side effects and therefore sustained drug action [12]. A study that was conducted by Machluf and coworkers [170] demonstrated that radio-labeled protein-loaded liposomes could be embedded within two membrane layers of a polymeric-based system such as calcium cross-linked alginate and alginate integrated with poly(l-lysine) for sustained release of radio-labeled bovine serum albumin both *in vitro* and *in vivo.* In another set of studies, it was postulated that the success of liposome release from polymeric-based systems could be due to mesh size of the matrix, size of liposome, diffusion, chemical, pH, and/or enzyme factor [8, 82, 112, 209]. In yet another study by Dhoot and Wheatley [168], liposome release from barium-alginate depots was reported to be influenced by the cross-linking ions. Leakiness of liposomes during the encapsulation process was due to high lipid content (i.e., cholesterol) during liposome fabrication for which a high liposomal escape was also observed. In comparing the liposome and degradable system to the liposome and nondegradable polymer-based systems, the results indicated that the liposomal release for the first system was due to degradation of the polymeric matrix, while for the second system an insufficient release was observed during the same period of study [210]. Nixon and Yeung [164] conducted a study together with Stenekes and coworkers [8] in which they could demonstrate that liposomes with low and high membrane fluidity were successfully released from a polymeric-based system in their intact form and with preserved size for approximately 60 days. Although pre-encapsulated drug-loaded liposome could show controlled drug release from the depot, majority of these studies have shown that the obtained drug release profiles depended to a greater extent on the liposomal burst effect rather than the diffusion process [11, 170, 201].

## 8. The Successes and Challenges Emerging from Composite Liposome and Polymeric-Based Technologies

The combination of liposome-based system and polymeric-based system for sustained release of therapeutically active compounds has been demonstrated to be successful in pharmaceutical applications. Sustained release profiles of different bioactive molecules such as gene, drugs, protein, and growth factor from liposome encapsulated in both natural or synthetic biodegradable polymeric material have been obtained [12, 169, 171]. The success of this drug delivery combination depends mostly on encapsulation efficacy and the type of drug release profile that is obtained. Efficiency in encapsulating drug-loaded liposome was reported to be dependent on several techniques, such as cross-linking agents (glutaraldehyde, formaldehyde, carbodiimide) or physical treatments (i.e., UV irradiation, freeze-drying), during fabrication process [152, 160]. Sustained release kinetics of the pre-encapsulated drug-loaded liposome depends most on the degradation rate of the polymeric materials. This system has added a remarkable advantage to both technologies (i.e., liposome-based and polymeric-based), though more so to the liposome technology since polymeric materials are more stable than liposomes. The following properties were achieved by embedding the liposomes into a polymeric-based system: (i) sustained release over prolonged periods of time, (ii) improved viscosity, (iii) stability of liposome, and (iv) improved half-life for both the drugs and liposome. In polymeric-based system incorporated with liposomes, drug delivery efficacy and preservation of drug bioactivity has been achieved. This is due to the fact that liposomes have a higher degree of biocompatibility when compared to polymeric materials [8, 36]. Although this composite system demonstrated improved success, there are still some major challenges that need to be overcome. Incorporation of toxic organic solvent or high heat during fabrication process can inhibit the activity of some bioactive molecules such as protein [11, 200]. Furthermore, since drug-loaded liposome release profiles seem to depend most on degradation of polymeric materials, majority of drug-loaded liposome may remain enmeshed within the depot or insufficient initial release at commencement of treatment may be a problem. At the same time, high overdose may occur during high degradation period. In either case, degradable polymeric material has demonstrated more efficacy than nondegradable polymeric material since with the latter depot, insufficient drug release was reported [210].

## 9. Future Perspective

Significant development has been reported on combination of the liposome-based technology with temporary depot polymeric-based technology in sustaining drug release over prolonged periods of time. However, combination of both drug delivery technologies into a single model of drug delivery has been reported to be associated with inadequate drug release. Since both materials can be easily manipulated, design of a new ideal temporary depot of the polymeric-based technologies to enhance therapeutic efficacy or improve the drug release profile is of a great interest. Integration of the more advanced types of liposome-based technologies such as targeted- or stimuli-sensitive liposomes in this system can enhance therapeutic efficacy. In addition, targeted liposome formulations, with targeted moieties such as antibodies, peptide, glycoprotein, polysaccharide, growth factors, carbohydrate, and receptors may increase liposomal drug accumulation in the tissues/cells via overexpressed receptors, antigen, and unregulated selectins. Sensitivity of liposomes to pH, light, magnetism, temperature, and ultrasonic waves can enhance therapeutic efficacy. Some polymeric systems have demonstrated some disadvantages in this application such as nondegradability that results in insufficient drug release. The use of a combination liposomal-based system with natural and/or synthetic polymeric biodegradable and/or nondegradable polymers may add strength to the depot while improving liposomal release profile. Although organic solvent are normally added during fabrication, nontoxicity should be rigorously assessed in *ex vivo* studies. In summary, the combination system, as a model of sustained release of drug-loaded liposome from temporary polymeric depots, has been declared successful but system improvements are demanded. Since this system is implantable, it may be useful in future for the management of chronic diseases such as Aid Dementia Complex, Tuberculosis, Cancer, or Neurodegenerative disorders, such as Parkinson's and Alzheimer's disease, which normally require regular doses over prolonged periods of time.
